# A Frequency Reconfigurable Folded Antenna for Cognitive Radio Communication

**DOI:** 10.3390/mi14030527

**Published:** 2023-02-24

**Authors:** Ahmed A. Ibrahim, Wael A. E. Ali, Moath Alathbah, Hesham A. Mohamed

**Affiliations:** 1 Electronics and Communications Engineering Department, Minia University, Minia 61519, Egypt; 2 Department of Electronics & Communications Engineering, College of Engineering and Technology, Arab Academy for Science, Technology and Maritime Transport (AASTMT), Alexandria 21937, Egypt; 3 College of Engineering, King Saud University, Riyadh 11451, Saudi Arabia; 4 School of Engineering, Cardiff University, Cardiff CF24 3AA, UK; 5 Electronics Research Institute, Microstrip Circuits Joseph Tito St, Huckstep, El Nozha, Cairo 11843, Egypt

**Keywords:** folded monopole antenna, cognitive radio, reconfigurable, PIN diode

## Abstract

In this work, a spectrum-sensing monopole antenna was used to operate in different frequency bands for cognitive radio applications. The proposed antenna consists of a folded monopole antenna with a partial ground plane, and it can be used for various wireless technologies operated at various frequencies from 1.5 to 3.5 GHz. The suggested antenna was printed on a RO4003 substrate with 3.38 permittivity and an overall size of 60 × 60 × 0.813 mm^3^. To achieve reconfigurability of the antenna, PIN diodes (HPND-4005) were inserted at different lengths along the antenna to obtain the desired performance. The antenna was fabricated and experimentally tested to validate the simulation outcomes, and distinct consistency between the simulation and measurement outcomes was obtained. Computer simulation tool (CST) software was used to design and simulate the suggested antenna and then the model was fabricated to validate the simulation outcomes.

## 1. Introduction

Emerging technologies pave the way for future applications with challenging requirements including miniaturization in size with improved performance. These applications need antennas with a wide bandwidth and a compact size to cover multiple frequency bands [1,2,3]. One of the main problems caused by increasing the number of users and their data rates is the scarcity of the spectrum and this can be solved by giving access to secondary users (SUs) to exploit the unoccupied channels of the primary users (PUs) using cognitive radio technology. Cognitive radio technology is considered as one of the most useful technologies that mitigates the problem of the unoccupied spectrum which in turn enhances spectral efficiency by carrying out dynamic spectrum management. This is considered a challenging task that needs accurate monitoring of PUs’ presence over the specified spectrum using spectrum sensing for different wireless applications [4,5]. These applications can cover various technologies such as GSM (1800 MHz, 1900 MHz), UMTS (2100 MHz), and WiFi (2400 MHz) [6,7,8].

Many efforts have been exerted to achieve multiband behaviour and many techniques have been employed to fulfill the demands of recent wireless communication technologies. Some of the employed techniques to achieve multiband behaviour are a fractal structure [9,10], a complementary split ring resonator metamaterial [11], a window grille cross-structure [12], a meandered offset-feed [13], a split ring resonator and inverted F slots [14], a bowtie slot patch [15], and DGS and DMS [16,17]. An additional capability that can be possibly integrated with the multiband behaviour of the antenna is the reconfigurability which has a vital role in the accomplishment of cognitive radio applications, and it can be carried out using PIN diodes [18,19,20,21], varactor diodes [22], lumped capacitors [23,24], and RF MEMS [25]. In [18], a semi-circular patch was loaded onto a triangular radiator with two inverted L-stubs to achieve reconfigurability between two frequency bands using a pair PIN diode. In order to achieve a triple-band reconfigurable antenna, a pair of PIN diodes are inserted on the two slots of a rectangular-shaped patch [19] for WLAN/WiMAX/ITU applications. In [20], a reconfigurable MIMO antenna was designed using FR4 substrate to switch between two band-stop filters at 1.77 and 4.75 GHz using a PIN diode with high isolation. Two PIN diodes were utilized in [21] to achieve reconfigurable notched band characteristics for a UWB antenna. Two band notched frequencies were reconfigured at 3.5 and 5.2 GHz for WiMAX/WLAN interference mitigation capability. Four varactor diodes were used in the introduced filtenna for cognitive radio applications [22]. The varactors’ tunning ranges from 3 GHz to 1.75 GHz when applying voltages from 9 V to 0 V, respectively. In [23], a reconfigurable triple-band antenna was implemented using a pair of lumped capacitors to obtain two resonance frequencies at 1.51 and 1.91 GHz besides the main resonance frequency of the patch at 2.45 GHz. The tunning range of the two lumped capacitors were 670 MHz and 990 MHz for the lower and higher generated frequencies, respectively, when different values of lumped capacitors were used (0.845 to 3.454 pF). A dual-band (2.4, 5 GHz) folded monopole antenna is presented in [24] for a WLAN application with a lumped capacitor on the front side for size reduction purposes. A UWB antenna with reconfigurable capability is presented in [25] for cognitive radio applications using six MEMS switches to obtain five frequencies in the C-band.

In this paper, we introduce a folded monopole antenna operating for various wireless applications. This can be achieved by embedding four PIN diodes on a multi-sectional antenna for reconfigurability purposes and the suggested antenna succeeded in resonating at the intended frequencies (1.45, 1.6, 2, 2.6, and 3.5 GHz) with reflection coefficient values less than −10 dB. The suggested antenna was designed, simulated, fabricated, and then tested to validate the achieved results and to confirm its suitability to operate for spectrum sensing in cognitive wireless applications. The return losses of the suggested antenna as well as the radiation patterns and gain values were obtained using CST 2019 software.

## 2. Folded Antenna

**Figure 1:**
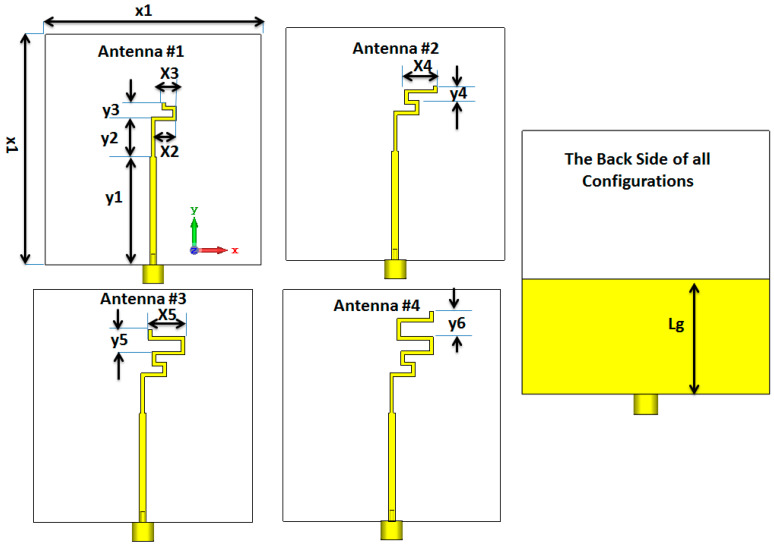
The evolution of the folded monopole antenna with x1 = 60 mm, x2 = 6 mm, X3 = 4 mm, x4 = 9 mm, x5 = 10 mm y1 = 30 mm, y2 = 11 mm, y3 = 4 mm, y4 = 4 mm, y5 = 6 mm, y6 = 7 mm, and Lg = 28 mm.

**Figure 2:**
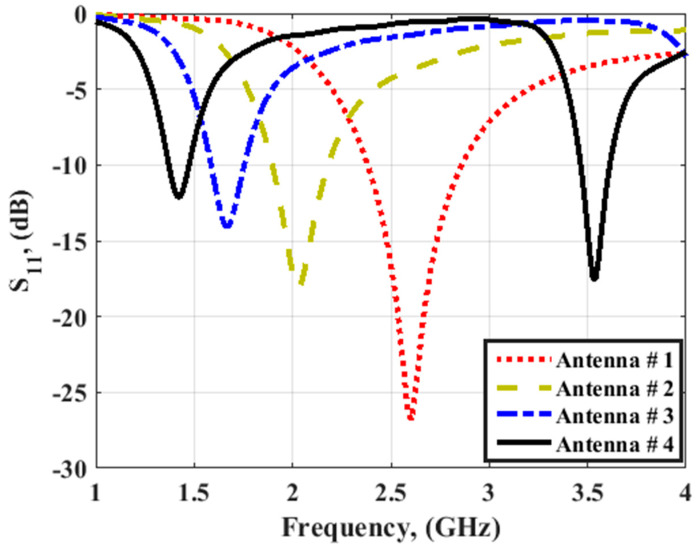
The simulated S_11_ outcomes for different antennas.

The design evolution of the suggested folded antenna is illustrated in Figure 1. The antenna is simulated on RO 4003 with a height of 0.813 mm and a dielectric constant of 3.38. Four cases of the antenna are simulated to produce the desired frequency bands. The antenna is a monopole antenna with a folded arm with a partially ground plane. A microstrip line of impedance 50 Ω was utilized to feed the antenna. The desired resonance frequency was achieved by changing the folded arm length as shown in Figure 1. Antenna #1 consists of two sections of folded arms to resonate at 2.6 GHz with a bandwidth extended from 2.45 to 2.85 GHz as illustrated in Figure 2 (the red dotted line). Antenna #2 achieved a frequency bandwidth from 1.9 GHz to 2.2 GHz with a central frequency of 2 GHz as illustrated in Figure 2 (the green dashed line). Antenna #3 is composed of many folded arms to reduce the operating frequency band to 1.6 GHz and bandwidth from 1.55 GHz to 1.75 GHz (the blue dotted dashed line). Finally, antenna #4 is operated at the fundamental mode of 1.45 GHz and the second mode is operated at 3.5 GHz with a bandwidth of (1.3–1.5 GHz) for the first band and (3.48–3.65 GHz) for the second band as illustrated in Figure 2 (the black solid line). 

**Figure 3:**
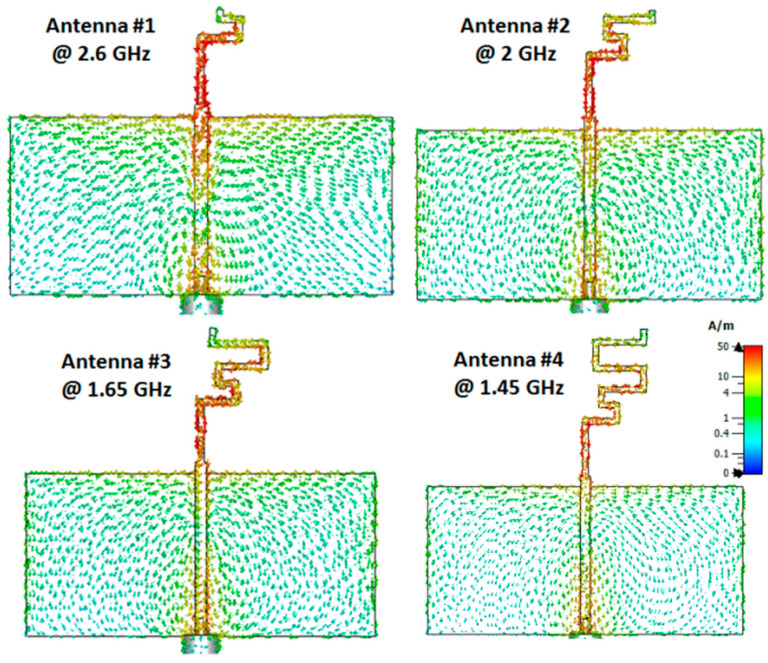
The simulated distributions of the surface current at the different frequency bands of the antennas.

**Figure 4:**
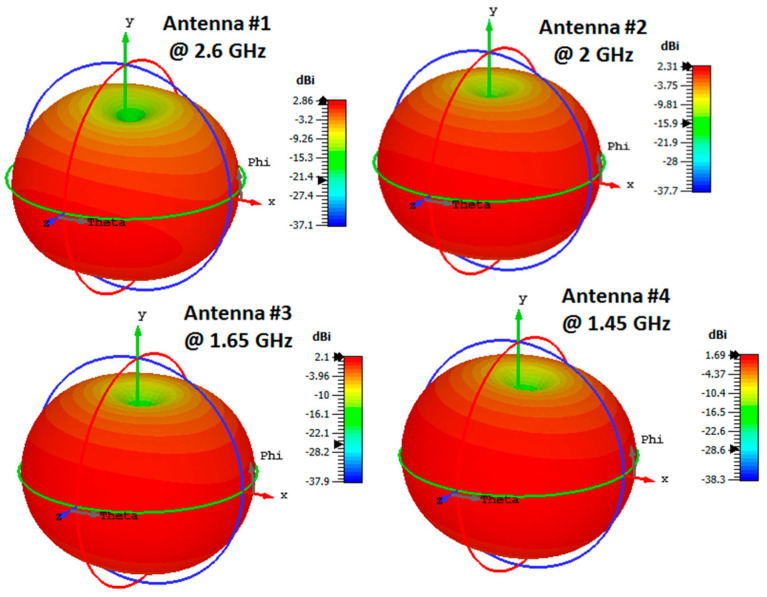
The simulated 3D radiation patterns at the different frequency bands of the antennas.

Figure 3 shows the distribution of the current of each case at different frequency bands. It can be seen that the current is collected around the folded arm which confirms the possibility of the antenna radiating effectively at the selected frequencies. The 3D radiation patterns for each case at different frequency bands are illustrated in Figure 4. The patterns are omnidirectional patterns at the desired frequency bands with around 2 dBi realized gain.

## 3. The Results of the Suggested Configuration

**Figure 5:**
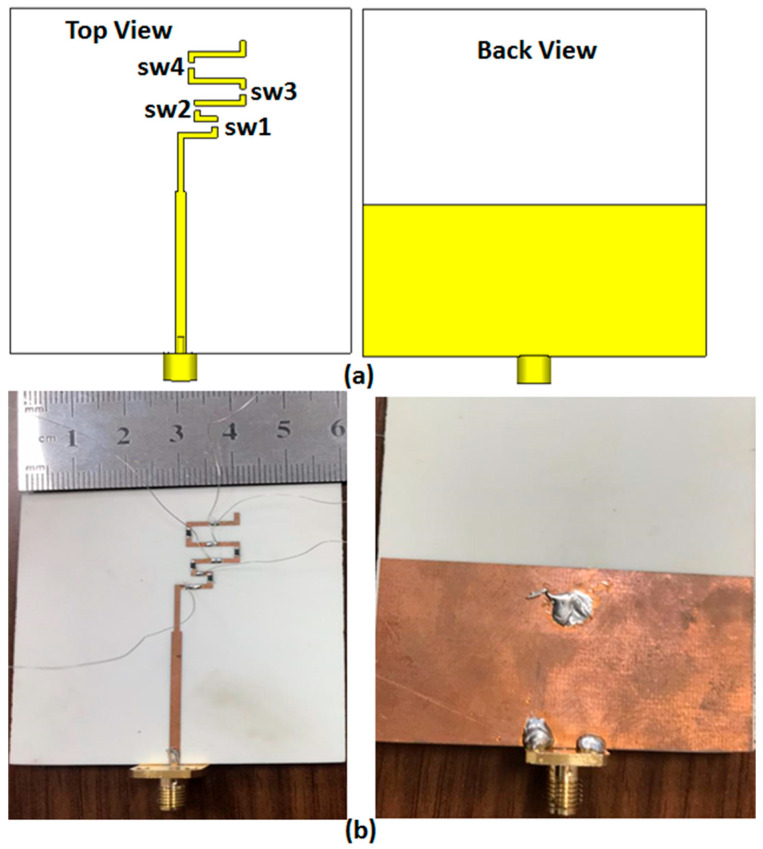
The suggested configuration of the antenna with four PIN diodes switches from sw1 to sw4: (**a**) the 2D layout and (**b**) the fabricated prototype.

To achieve the frequency reconfigurability of the folded antenna, the four switches (PIN diodes HPND-4005, Broadcom Inc., San Jose, CA, USA) were used to connect or disconnect between the arms as presented in Figure 5a. The photo of the fabricated prototype is illustrated in Figure 5b. An external DC voltage was applied to activate the PIN diodes. The R&S ZVB 20 (Rohde & Schwarz, Munich, Germany) vector network analyzer (VNA) was used in the measurement.

**Figure 6:**
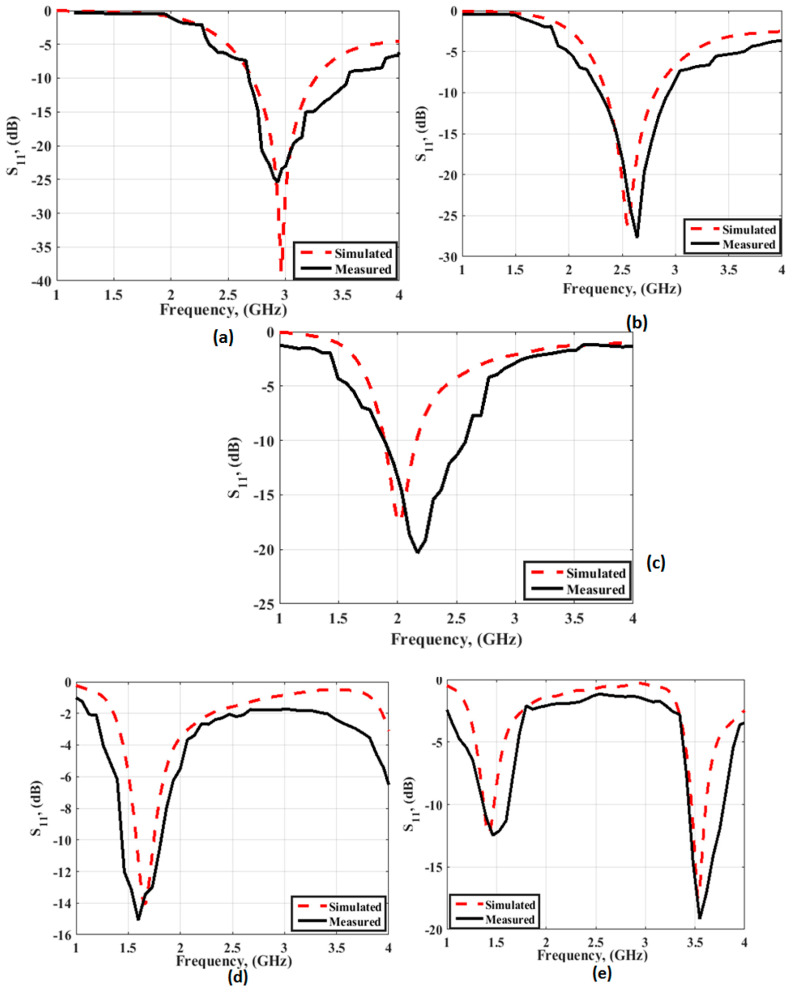
The S_11_ simulation and measurement outcomes of the folded antenna with PIN diodes: (**a**) all of the switches off, (**b**) sw1 on, (**c**) sw1 and sw2 on, (**d**) sw1, sw2, and sw3 on, and (**e**) all of the switches on.

Figure 6 depicts the simulated and tested reflection coefficient outcomes of the folded model in various cases of the four PIN diodes. Figure 6a shows the antenna outcomes when all of the switches were off. The tested results show that the antenna operated at a center frequency of 3 GHz with a bandwidth from 2.6 GHz to 3.5 GHz. When sw1 was on, the antenna worked at 2.6 GHz with a bandwidth from 2.4 GHz to 3 GHz as illustrated in Figure 6b. When sw1 and sw2 were on, the antenna operated at 2.2 GHz with a bandwidth extended from 1.9 GHz to 2.55 GHz as shown in Figure 6c. Figure 6d illustrates that the antenna operated at a center frequency of 1.55 GHz with a bandwidth from 1.44 GHz to 1.8 GHz when the three switches were on.

Finally, when all of the switches were on, as shown in Figure 6e, there was a dual-band at 1.5 GHz and a second mode at 3.5 GHz with a bandwidth from 1.3 GHz to 1.6 GHz for the first band and from 2.48 GHz to 3.75 GHz for the second band. As well, the two outcomes have a good match with a small discrepancy between them. This is due to the fabrication and measurement process that cannot be tackled.

**Figure 7:**
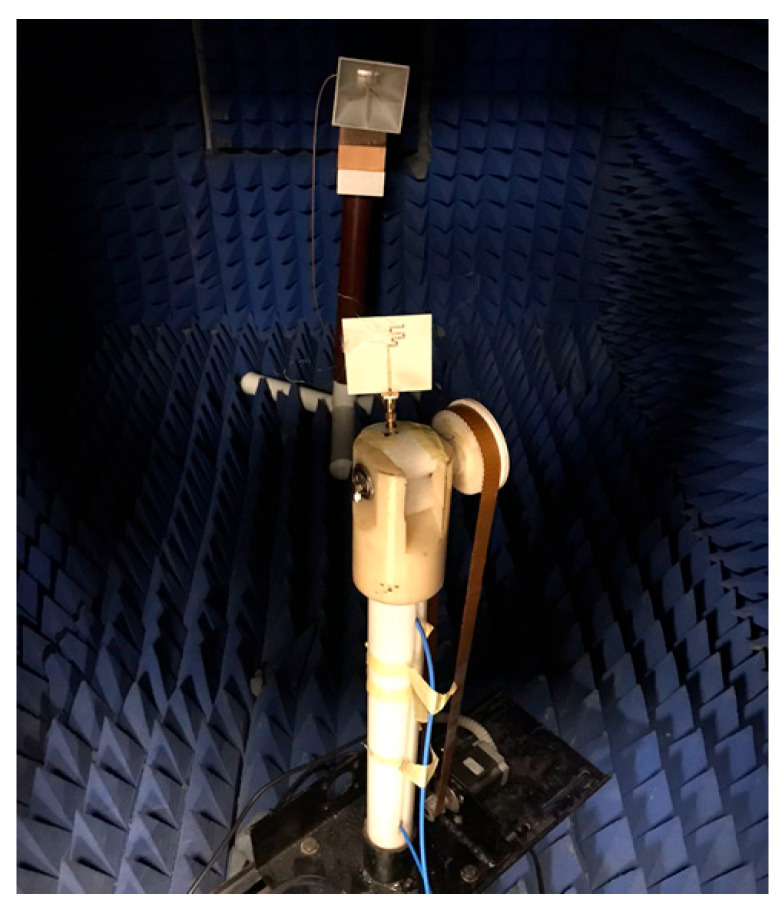
The setup of the radiation pattern photo.

**Figure 8:**
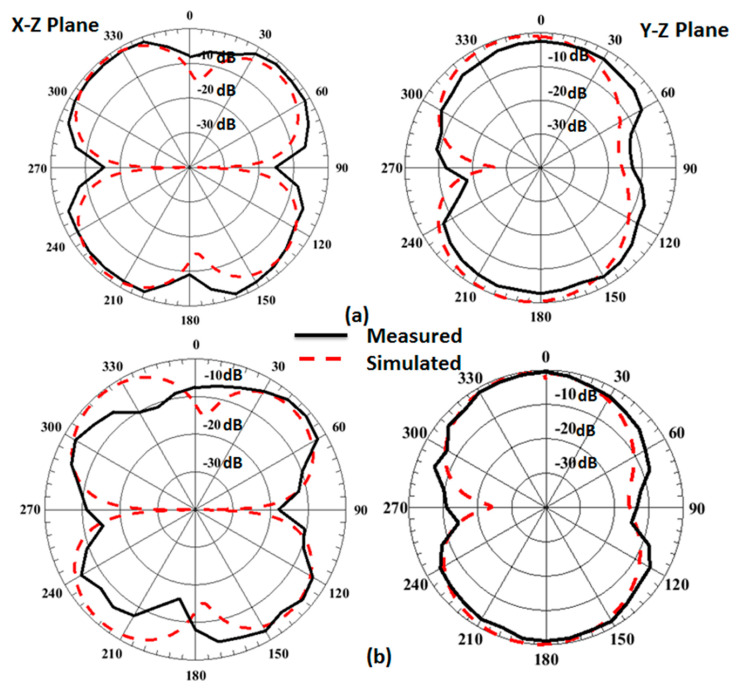

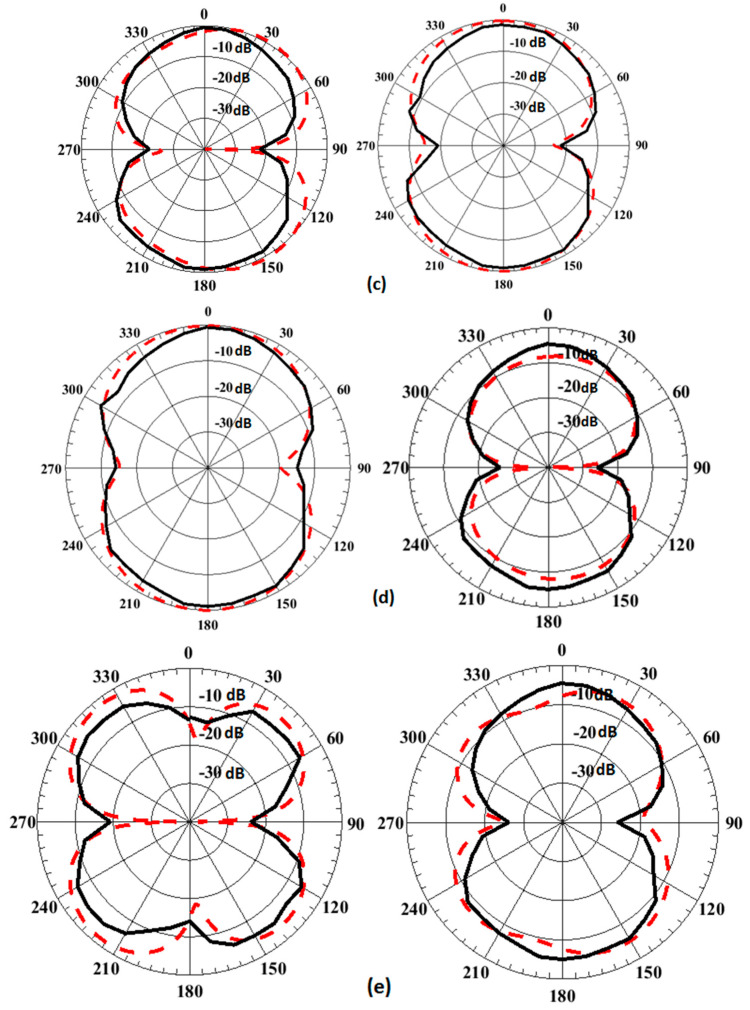
The normalized 2D patterns of the antenna at φ = 0° and φ = 90°: (**a**) at 3 GHz (all off), (**b**) at 2.6 GHz sw1 on, (**c**) at 1.6 GHz sw1, sw2, and sw3 on, (**d**) at 1.5 GHz (all on), and (**e**) at 3.5 GHz (all on).

The folded antenna was placed inside the anechoic chamber to test the radiation characteristics of the suggested antenna as illustrated in Figure 7. The 2D normalized tested and simulated radiation patterns at φ = 0° (X-Z plane) and φ = 90° (Y-Z plane) at different frequency bands based on the switch’s states are shown in Figure 8. The antenna has a bidirectional pattern at φ = 90° and semi-omnidirectional at φ = 0°. In addition, the two outcomes have a good match with a small shift between them. This is because of the measuring process.

The proposed design was compared to other works and is tabulated in Table 1. Table 1 illustrates that the suggested antenna has good performance which suggests that it should be used in cognitive radio applications.

## 4. Conclusions

A folded microstrip-fed monopole antenna for cognitive radio applications has been demonstrated. The proposed antenna has been designed for operation at various frequencies from 1.5 to 3.5 GHz with an overall size of 60 × 60 × 0.813 mm^3^. The reconfigurability behaviour was achieved by inserting four PIN diodes at different lengths along the antenna to resonate at 1.45, 1.6, 2, 2.6, and 3.5 GHz with reflection coefficient values less than −10 dB. The antenna was fabricated and experimentally tested to validate the simulation outcomes with consistency between the outcomes being illustrated.

## Figures and Tables

**Table 1:** Comparison between reported antennas and our work.

Ref.	ε_r_/h (mm)	Size (mm^2^)	f_o_ (GHz)	No.# of Freqs.	Actuators
[18]	2.1/0.254	40 × 50	1.8, 2.1	2	2 PIN diodes
[19]	4.4/1.6	20 × 20	3.6, 5.5, 8.1	3	2 PIN diodes
[20]	4.4/1.6	48 × 24	1.77, 4.75	2	1 PIN diode
[21]	4.5/1.524	17 × 23	3.5, 5.2	2	2 PIN diodes
[22]	3.38/0.813	80 × 80	2.16, 2.8, 3	3	4 varactor diodes
[23]	2.2/1.6	80 × 80	1.51, 1.91, 2.45	3	2 lumped capacitors
[24]	4.4/0.8	65 × 100	2.4, 5	2	1 lumped capacitor
[25]	4.4/1.6	40 × 40	4, 5.6, 5.8, 7.2, 7.8	5	6 MEMS
This work	3.38/0.813	60 × 60	1.5–3.5, 1.6, 2, 2.5, 3	6	4 PIN diodes

## Data Availability

All data generated or analyzed during this study are included in the article.
